# Design and Fabrication of a Piezoelectric Bimorph Microphone with High Reliability and Dynamic Range Based on Al_0.8_Sc_0.2_N

**DOI:** 10.3390/mi16020186

**Published:** 2025-02-04

**Authors:** Ruixiang Yan, Yucheng Ji, Anyuan Liu, Lei Wang, Songsong Zhang

**Affiliations:** 1 School of Microelectronics, Shanghai University, Shanghai 200444, China; yan2000829@126.com (R.Y.);; 2 Chengdu Chimesen Tech. Inc., Chengdu 610000, China; wanglei@chimesen.com

**Keywords:** piezoelectric bimorph microphone, Al 0.8 Sc 0.2N, high robustness, performance and dynamic range, acousitic overload point, signal-to-noise ratio, microphone theoretical model, imapct test

## Abstract

With the development of technology, MEMS microphones, which are small-sized and highly uniform, have been applied extensively. To improve their reliability in extreme environment and overcome the constraints of traditional microphones, this article presents a piezoelectric bimorph MEMS microphone using ${\mathrm{Al}}_{0.8}{\mathrm{Sc}}_{0.2}N$. In the article, the high robustness of piezoelectric microphones and the reasons for choosing ${\mathrm{Al}}_{0.8}{\mathrm{Sc}}_{0.2}N$ as piezoelectric materials are described. The sensitivity of an ${\mathrm{Al}}_{0.8}{\mathrm{Sc}}_{0.2}N$-based piezoelectric bimorph compared with the traditional structure are revealed through FEA. Subsequently, a lumped element microphone model is constructed and all noise sources are evaluated comprehensively. The difference in output noise caused by different structures is calculated. The designed piezoelectric microphone, which comprises eight triangular cantilever beams, was fabricated on a chip with an area of 900 μm × 900 μm. The sensitivity of the designed microphone achieves 1.68 mV/Pa, with a noise floor of −110 dBA and SNR of 54.5 dB. The acoustic overload point of the microphone stands at 147 dB SPL, and following the impact test, the survival rate was 100%. Compared to traditional MEMS microphones, the microphone achieves a dynamic range of 107.5 dB.

## 1. Introduction

**Figure 1:**
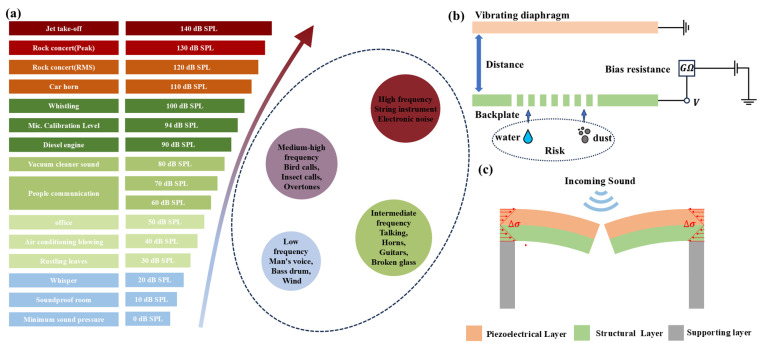
(**a**) Sounds with varying frequency and SPL show in diverse forms in our daily lives. (**b**) Working principle of capacitive MEMS microphone. (**c**) Working principle of piezoelectric microphone.

Sound is mainly generated by the vibration of objects and is then transmission through media, including air, solids, and liquids. Finally, these sound waves are sensed and interpreted by human ears [1]. The fundamental characteristics of sound are shaped by two elements: frequency and sound pressure level (SPL). Specifically, Frequency of sound wave determines its pitch and tone color, with the audible frequency range extending from 20 Hz to 20 kHz [2]. SPL is a physical quantity representing the intensity of sound. Under standard atmospheric pressure conditions, its maximum is 194 dB [3]. As depicted in the Figure 1a, these factors interact and mold the sounds we perceive.

In order to collect sound, microphones have been designed. Typically, a microphone’s performance is evaluated based on its ability to operate without distortion, specifically its ability to capture the full frequency and SPL ranges of the sound [4]. Commonly used microphones include electret microphones (ECMs) and condenser microphones. ECMs have the advantages of high sensitivity, compact size and low cost, but they also have the disadvantages of being sensitive to ambient noise, being affected by humidity and temperature, and having weak electromagnetic interference resistance [5]. Condenser microphones have the advantages of high sensitivity, wide frequency response, and accurate sound reproduction, but they are costly, fragile, require external power, and are sensitive to handling noise [5]. With the advent of micro-electro-mechanical system (MEMS) technology in the late 1980s, MEMS devices possess the advantages of small size, light weight, and high reliability, thereby rendering the development of MEMS microphones an increasing trend [6].

Nowadays, MEMS microphones have become ubiquitous in electronic products [7]. In comparison with ECMs, MEMS microphones possess several advantages over ECM microphones, including a smaller size, superior integration with semiconductor technology, higher reliability, lower power consumption, and enhanced resistance to environmental factors such as temperature and humidity [8]. Furthermore, the cost-effectiveness of MEMS microphones stems from their manufacturing process, which utilizes silicon wafer deposition technology [6]. In recent years, voice control has become the important sales market for MEMS microphones. Sound acquisition has also appeared in new application scenarios, including wearables and the Internet of Things, which bring challenges to the MEMS microphones [9]. As shown in Figure 1a, in specific scenarios, such as directed sound capture at rock concerts, microphones operate in extremely high sound pressure environments, which necessitates a high acoustic overload point (AOP) and a wide dynamic range to prevent failures [10,11]. In the environment of the Internet of Things (IoT), users often need to utilize microphones to capture faint sounds, thereby posing specific requirements for reducing the noise floor of these microphones [12]. Certain scenarios also necessitate waterproof performance for MEMS microphones. In all scenarios, MEMS microphones require features of high performance, high robustness, and wide dynamic range.

MEMS microphones can be designed by various principles, such as the piezoelectric effect, piezoresistive effect, optical effect, and capacitive effect [13,14,15,16,17,18]. Among MEMS microphones, MEMS capacitive microphones have achieved commercialization, while piezoelectric MEMS microphones have garnered significant attention. Regardless of the sensing technology employed, MEMS microphones share fundamental parameters such as sensitivity, signal-to-noise ratio (SNR), dynamic range and bandwidth, which collectively dictate their performance.

The sensing structure of MEMS capacitive microphones usually consists of a flexible diaphragm and a stationary backplate [18]. The working principle is shown in Figure 1b. Due to the large bias resistance, the time constant is large, enabling capacitors to operate in a constant charge mode [19]. When acoustic pressure acts upon the diaphragm, it induces a change in the parallel plate distance. As the parallel plate capacitor operates in the constant charge mode, it ultimately causes a voltage variation.

Although MEMS capacitive microphones have achieved success in the market, they also have their inherent limitations [6]. During operation, they necessitate the maintenance of an immaculate space between the plates. Any ingress of dust or water into the gap will exert an adverse impact on the microphone’s performance and may even lead to malfunctions [20]. In addition, for the most classic structure of MEMS capacitor microphones, the deflection of the clamped diaphragm $\omega \left(r\right)$ is given by [21].

(1) $$\omega \left(r\right)=x{〔1-{\left(\frac{r}{a}\right)}^{2}〕}^{2}$$

where *x* is the central displacement of the diaphragm, *a* is the radius, and *r* is the distance along the radius. This results in variations in diaphragm displacement across different locations, leading to a sharp increase in nonlinearity under high SPL [22]. Additionally, capacitive microphones are susceptible to the pull-in effect, which can lead to device failure [23]. These limitations hinder their deployment in extreme environments.

Piezoelectric microphones offer a solution to enhance the reliability of MEMS microphones, as they exhibit superior reliability advantages [24]. Their working principle is shown in Figure 1c. They use the stress generated in the bending cantilever beam to generate electrical signals [25]. There is no gap between the sensing structures of the piezoelectric microphone, reducing the risk of failures [26]. In addition, Patel et al. mentioned that for piezoelectric materials, the nonlinearity in the piezoelectric effect is given by the equations [27]:

(2) $$\sigma_{p}^{11}=E_{p}\varepsilon_{p}^{11}+\frac{\mu_{1}}{2}{\left(\varepsilon_{p}^{11}\right)}^{2}-E_{p}d_{31}E_{\mathrm{field}}-\mu_{2}\varepsilon_{p}^{11}E_{\mathrm{field}}$$

(3) $$Q=d_{31}\sigma_{p}^{11}$$

where $E_{p}$ is the Young’s modulus, $E_{\mathrm{field}}$ is the electric field strength, and $d_{31}$ is the piezoelectric constant. Equation (2) relates the axial stress $\sigma_{p}^{11}$ to the axial strain $\varepsilon_{p}^{11}$. $\mu_{1}$ and $\mu_{2}$ represent piezoelectric nonlinearity. For piezoelectric cantilevers under small deformation conditions, $\mu_{1},\mu_{2},\varepsilon_{p}^{11}$ are usually of an order of magnitude, but several orders of magnitude smaller than $E_{p}$, making the first term on the right-hand side of Equation (2) important. Ultimately, according to Equations (2) and (3), the degree of nonlinearity in the output charge *Q* is minimal. As a result, this characteristic endows piezoelectric microphones with a higher AOP compared to capacitive microphones [27]. Compared with MEMS capacitive microphones, the piezoelectric microphone exhibits high robustness, constituting a significant advantage [28].

Piezoelectric microphones generally exhibit limited advantages in terms of sensitivity and noise floor [24]. Therefore, this article aims to explore methods for enhancing these performance metrics to the greatest extent possible, so as to achieve a microphone with high reliability and a high dynamic range even under high sound pressure level environmental conditions.

## 2. Design, Simulate, and Fabricate Devices

### 2.1. Selection of Piezoelectric Materials

The performance of piezoelectric microphones is influenced by the choice of piezoelectric materials. Among the most commonly utilized piezoelectric materials, zinc oxide (ZnO), lead zirconate titanate (PZT), and aluminum nitride (AlN) each possess distinct characteristics. In comparison to the fabrication challenges associated with other materials, aluminum nitride (AlN) has garnered significant attention due to its compatibility with CMOS technology, which facilitates integration and manufacturing processes [29,30]. The common piezoelectric material coefficients are given in Table 1 [31,32,33,34,35].

While the piezoelectric coefficient $d_{31}$ of aluminum nitride (AlN) may not be the highest among piezoelectric materials, there are strategies to enhance this property. Recently, researchers have discovered that the incorporation of scandium (Sc) into AlN can improve its piezoelectric characteristics, leading to the development of materials with properties similar to those of ${\mathrm{Al}}_{0.8}{\mathrm{Sc}}_{0.2}N$ [35]. The loss angle only increases slightly [36]. Seo et al. proposed that the theoretical maximum signal-to-noise ratio of the piezoelectric microphone is influenced by piezoelectric materials [37]. It is related to several properties of the piezoelectric materials, and these properties determine the maximum theoretical electromechanical conversion efficiency of the piezoelectric microphone. The relationship between SNR and relevant material properties is shown in Formula (4), and the result of Formula (4) can be regarded as a potential optimization parameter so as to select more excellent piezoelectric materials [37]. The specific relationships are as follows:

(4) $${SNR}^{2}\propto \frac{d_{31}^{2}}{\varepsilon_{r}tan\delta }$$

The optimization parameter can be computed from the data in Table 1 and Formula (4). It is evident that by employing ${\mathrm{Al}}_{0.8}{\mathrm{Sc}}_{0.2}N$ as a piezoelectric material, which is possible to obtain a higher SNR. While theoretically increasing the scandium (Sc) content as much as possible may seem beneficial for microphones, there exists a threshold beyond which the crystal structure undergoes changes, leading to the loss of the piezoelectric effect. Therefore, an optimal Sc content must be determined to balance performance and material stability [38]. In addition, the increase of Sc content will bring new challenges to the process. ${\mathrm{Al}}_{0.8}{\mathrm{Sc}}_{0.2}N$, which is stable in the existing process technology, is selected to prepare the microphone with high performance, reliability and dynamic range.

### 2.2. Principle of Device Design

A piezoelectric microphone serves as a device that converts sound pressure into electrical signals. Its performance hinges on its capacity to effectively transform sound pressure into electrical signals. To optimize the design of this device, it is crucial to comprehend its operational principles and design foundations.

Here, we discuss the triangular cantilever beams commonly used in piezoelectric microphones, taking the commercially available Vesper product as an illustrative example [6]. This is because the stress distribution of the triangular cantilever beam is more advantageous. The stress gradually increases from the top of the triangle to the bottom. In addition, the processing difficulty is relatively lower. Du et al. have deduced the stress analysis of rectangular cantilever beam [39]. This is also applicable to the stress analysis of triangular cantilever beam, so we will use a similar method for discussion.

**Figure 2:**
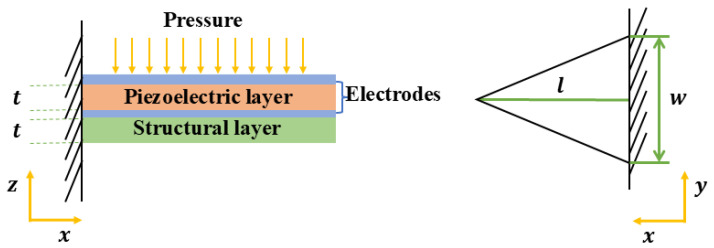
Structure of the Cantilever Beam of a Piezoelectric Microphone.

As shown in Figure 2, a piezoelectric cantilever typically consists of a piezoelectric layer, a structural layer, and electrode layers. If the electrode layer is very thick, it will cause great stress loss in the piezoelectric layer. For the purpose of simplifying the analysis, it is assumed that the electrode layers are negligible in thickness, and both the piezoelectric layer and the structural layer possess identical stiffness and thickness. Consequently, the electrodes can be disregarded, and the neutral axis is presumed to lie at the center. *t* is the thickness of the piezoelectric layer and the structural layer, *l* is the length of the beam, and *w* is the length of the triangle’s base.

When the cantilever beam bends under the sound pressure, the stress in the piezoelectric layer generates electric charge. For a cantilever, the charge generated $Q_{\left(x,y,z\right)}$ is mainly caused by the stress in the *x* direction.

(5) $$Q_{\left(x,y,z\right)}=d_{31}\sigma_{\left(x,y,z\right)}$$

where $\sigma_{\left(x,y,z\right)}$ is the in-plane stress. The most important thing is to calculate the stress $\sigma_{\left(x,y,z\right)}$ of the beam. Given that the microphone operates within its working environment, the deflection of the cantilever beam is smaller than the length of the cantilever beam. This problem can be effectively analyzed by applying the Euler-Bernoulli beam theory [40]. First, based on the approximate relationship between the displacement ω(x) and the applied external force, the displacement ω(x) is given by [41]:

(6) $$\omega \left(x\right)=\frac{1}{24}Ax^{4}-\frac{1}{6}Alx^{3}+\frac{1}{4}Al^{2}x^{2}$$

where $A=\frac{q}{\mathrm{EI}}$, q is the external force per unit length (N/m), E and I respectively represent the Young’s modulus and inertia moment of the beam. In this manner, ω(x) can be determined, and by employing the Euler-Bernoulli beam theory, $\sigma_{\left(x,y,z\right)}$ can be determined [40]:

(7) $$\sigma_{\left(x,y,z\right)}=-zE\frac{d^{2}\omega \left(x\right)}{dx^{2}}$$

where *z* is the distance from the neutral axis. Finally, the sensitivity *V* can be expressed as:

(8) $$V=\frac{Q}{C}=\frac{\int d_{31}\sigma_{\left(x,y,z\right)}}{C}$$

where *Q* and *C* are the total charge and capacitance of the device, respectively. The calculation results for the sensitivity of a piezoelectric microphone can indeed be intricate. However, these can be simplified under certain conditions. When identical materials are utilized, the sensitivity achieved is directly proportional to specific geometric parameters:

(9) $$V\propto \frac{l^{2}\cdot w}{t^{2}}$$

Therefore, adjusting geometric parameters can lead to higher sensitivity. However, sensitivity is not the sole criterion for evaluating microphones. As mentioned previously, parameters such as capacitance, frequency response, and area also play crucial roles in determining the performance of microphones [42]. For example, if the capacitance is too small, the electrical output is attenuated by parasitic capacitance; if the 1st resonance frequency is too low, the working band narrows; if the area is too large, MEMS microphones lose miniaturization advantage. Therefore, these properties need to be considered jointly [6]. The figure of merit (FOM) ψ is proposed here to discuss the combined influence of the aforementioned parameters. The FOM ψ is given by:

(10) $$\psi =\frac{V_{\mathrm{out}}^{2}\cdot C_{p}\cdot f_{\mathrm{res}}^{2}}{A}$$

where $V_{\mathrm{out}}$ is the sensitivity of 1 kHz when the microphone is under 1 Pa RMS sound pressure, $C_{p}$ is the capacitance, $f_{\mathrm{res}}$ is the 1st resonance frequency, and A is the area. The Equation(10) nullifies the impact of area dimensions on the device structure. Notably, the thickness parameter is not introduced. This is due to the fact that any alteration in the thickness of the piezoelectric material would directly lead to a change in the level of process complexity [43].

**Figure 3:**
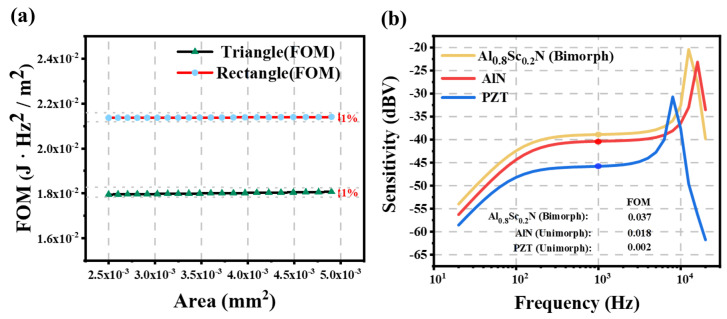
(**a**) FOM of triangular and rectangular beam exposed to 1 Pa RMS sound pressure. (**b**) Sensitivity and FOM of triangular cantilever beam of different materials and structures.

The finite element analysis (FEA) was employed to simulate the figure of merit (FOM) of different cantilever beams with varying areas but identical thickness. The results are presented in Figure 3a. The fluctuation of the obtained FOM is less than 1%, which can be ascribed to the error margin of the FEA. This indicates that, for these structures, merely altering the area size does not enhance the key performance. While rectangular cantilever beams offer theoretically superior performance, the manufacturing challenges posed by beam curling during processing render them a less common structure [44]. The Equation (10) normalizes the microphone parameters by area, allowing for a comparison of the relative merits of different designs. This approach provides a direction for exploring the advantages of various structures.

Previously, the majority of piezoelectric microphones adopted a unimorph structure. Nowadays, an increasing number of researchers and designers are opting for the bimorph structures. The distinct advantage of the bimorph structures lies in its ability to position the neutral axis more effectively and utilize the stress within its layers [45,46]. Under the condition of almost constant sensitivity, the output energy is increased, which has been proved to be able to improve the SNR of the device [24].

The sensitivity of bimorph ${\mathrm{Al}}_{0.8}{\mathrm{Sc}}_{0.2}N$ and unimorph PZT and AlN microphone with the same condition is simulated, and the FOM are calculated. As can be seen from Figure 3b, despite PZT possessing the highest piezoelectric coefficient, its sensitivity is significantly reduced because of its larger dielectric constant. Furthermore, under identical conditions, the stiffness of PZT results in a very low 1st resonance frequency for PZT microphones, thereby impacting its working bandwidth. On the other hand, microphones utilizing ${\mathrm{Al}}_{0.8}{\mathrm{Sc}}_{0.2}N$ as the piezoelectric layer exhibit a sensitivity that is 1.46 dBV higher than those using AlN as the piezoelectric material. This translates to an impressive 18% increase in sensitivity. At the same time, due to the bimorph structure, the capacitance and output energy are doubled, thereby contributing to the enhancement of the SNR of the device. [24]. In general, the use of ${\mathrm{Al}}_{0.8}{\mathrm{Sc}}_{0.2}N$ as a piezoelectric material results in the highest FOM. In this paper, we adopt ${\mathrm{Al}}_{0.8}{\mathrm{Sc}}_{0.2}N$ as piezoelectric material and utilize bimorph structure to improve the performance, reliability and dynamic range of the microphone.

### 2.3. Noise

While FEA effectively and conveniently predicts the sensitivity of the device, noise, a critical performance parameter, typically requires theoretical calculation [47]. This section focuses on the primary noise sources in piezoelectric microphones, conducting an in-depth analysis of the impact of bimorph structures on noise characteristics.

The most common source of inherent noise in piezoelectric microphones is thermal noise, determined by Nyquist relation [48]. In this theory, the noise source is regarded as a voltage source in series with the pure resistor, and its power spectral density $V_{R_{ep}}^{n}$ is:

(11) $$V_{R_{ep}}^{n}=4k_{b}TR_{ep}$$

where $k_{b}$ is the Boltzmann constant, *T* is the Kelvin temperature, and $R_{ep}$ is the resistance. Other types of noise can be computed through the construction of an analogous circuit model [49]. Whether it is electrical noise, mechanical noise, or acoustic noise, it can be calculated by Nyquist relation, and the units are $V^{2}/\mathrm{Hz}$, $m^{3}\cdot s^{-1}/\mathrm{Hz}$, ${\mathrm{Pa}}^{2}/\mathrm{Hz}$, respectively.

**Figure 4:**
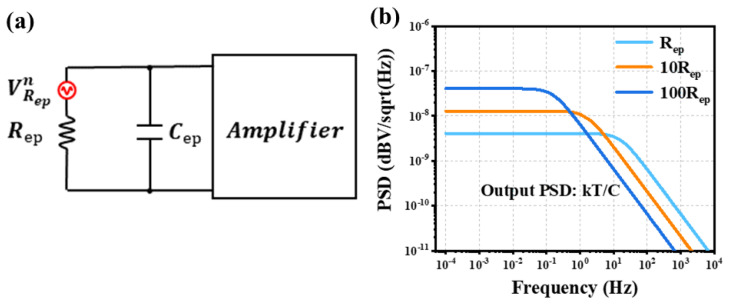
(**a**) The equivalent circuits of piezoelectric materials. (**b**) The low-pass filtering of thermal noise.

First, the inherent noise of piezoelectric materials will be discussed. Piezoelectric materials are modeled as a parallel combination of a resistor and a capacitor, as depicted in Figure 4a. The relationship between these two elements is given by Equation (12) [50]. The resistance is inversely related to the loss angle. A larger loss angle means smaller resistance and less thermal noise generation. However, as shown in Figure 4b, the combination of the resistance and capacitor constitutes a low-pass filter. The noise spectral density, derivable from Equation (13), is directly proportional to the loss angle and inversely proportional to the capacitance. When the power spectral density (PSD) of the output noise is integrated across infinite frequency bands, the resultant value is independent of the resistance. This type of noise is termed $\frac{k_{b}T}{C}$ noise [48].

(12) $$R_{ep}=\frac{1}{\omega \cdot tan\delta \cdot C_{ep}}$$

(13) $$V_{noise}^{out}=\frac{\sqrt{4k_{b}TR_{ep}}}{1+j\omega R_{ep}C_{ep}}=-\sqrt{\frac{4k_{b}Ttan\delta }{\omega C_{ep}}}$$

**Figure 5:**
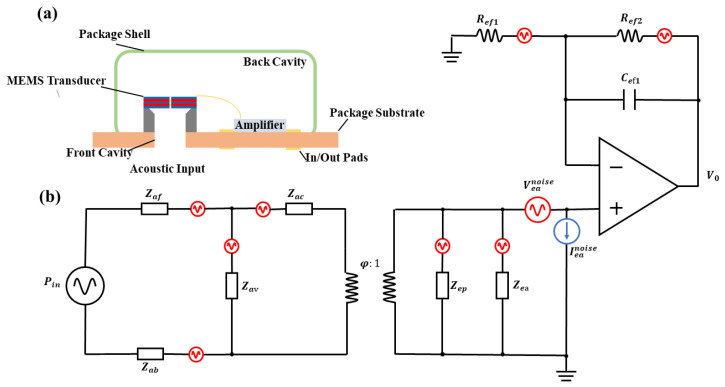
(**a**) The entire structure of the MEMS microphone. (**b**) The lumped element model of the MEMS microphone.

The total noise in a microphone encompasses not only the thermal noise of piezoelectric materials, but also acoustic noise, mechanical noise, and others. These noise sources are linked to the microphone’s overall structure. The structure of the actual piezoelectric microphone is shown in Figure 5a. A conventional microphone system consists of three fundamental constituents: a MEMS sensor, an amplifier employed for signal conditioning and amplification, and an enclosure serving as a shield to impede the ingress of sound pressure from the rear. With regard to this microphone packaging model, a lumped-element model can be formulated to evaluate the influence of each component on the noise performance of the device.

The lumped element model is presented in Figure 5b, where each element corresponding to those in Figure 5a can be identified. The lumped elements of each primary component are defined based on the fundamental physical properties of the respective component. To preclude ambiguity, the naming convention adopted here dictates that the first sub-script of an element denotes the domain (acoustic, denoted as ‘a’, or electrical, denoted as ‘e’), while the second sub-script offers identification (front cavity, denoted as ‘f’, or back cavity, denoted as ‘b’).For instance, $Z_{af}$ denotes the lumped impedance of the front cavity within the acoustic domain, which is composed of the acoustic compliance, acoustic mass, and acoustic resistance at that specific point. The parameter φ signifies the capacity of the cantilever to transform stress into electric charge. $Z_{ea}$, $V_{ea}^{\mathrm{noise}}$, and $I_{\mathrm{ea}}^{\mathrm{noise}}$ represent the impedance, voltage noise, and current noise of the amplifier respectively. Their values are calculated in accordance with the research work of Horowitz [49].

**Figure 6:**
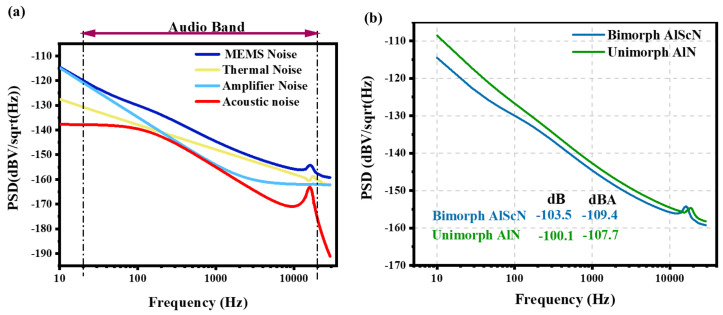
(**a**) Theoretical Power Spectral Density (PSD) of each noise component of MEMS microphones in the audio band. (**b**) Total noise of piezoelectric microphones with different structures and materials.

Based on the lumped element model and Equation (11), the contributions and trends of each noise component to the total noise are calculated, as illustrated in Figure 6a. Here, the noise generated by the lumped elements in the acoustic domain is denoted as acoustic noise. In the low-frequency regime, the noise is mainly originated from the amplifier. This is because the impedance of the MEMS microphone is extremely high, which amplifies the influence of the amplifier’s current noise on the overall noise level. Beyond this frequency range, the dominant noise source becomes the material losses within the piezoelectric materials. It is worth noting that the acoustic noise experiences a significant surge only at the resonance frequency of the piezoelectric microphone, yet its contribution to the total noise remains relatively small. Generally, the noise from microphone materials accounts for more than 40% of the total noise. Therefore, optimizing noise at the MEMS level is of crucial significance.

Based on the parameters presented in Table 1, the noise performance of a bimorph ${\mathrm{Al}}_{0.8}{\mathrm{Sc}}_{0.2}N$ and a unimorph AlN microphone is simulated under identical geometric conditions, as depicted in Figure 6b. Evidently, even when ${\mathrm{Al}}_{0.8}{\mathrm{Sc}}_{0.2}N$, a piezoelectric material with a relatively higher loss angle, is employed, the bimorph structure results in lower noise levels due to its larger capacitance. Within the audio frequency band, the noise level of the bimorph microphone measures −109.4 dBA, while that of the unimorph microphone reaches −107.7 dBA. This disparity becomes more pronounced as the device’s capacitance increases. Consequently, the utilization of a bimorph structure holds promise for mitigating the noise in piezoelectric microphones.

### 2.4. Fabrication and Characterization

**Figure 7:**
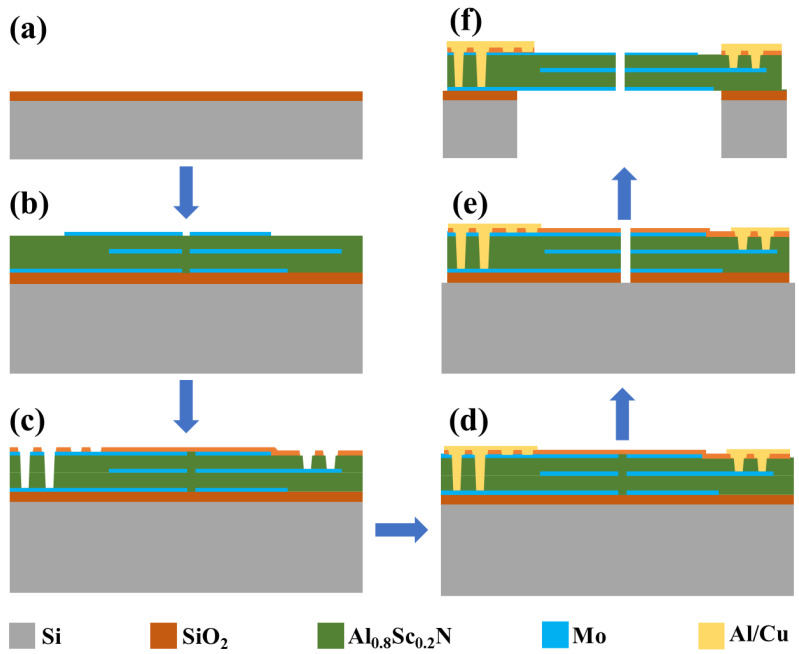
Fabrication process flow of the MEMS piezoelectric microphone.

The designed MEMS microphone is fabricated by the process flow shown in Figure 7. First, as shown in Figure 7a, a layer of thermal oxide is grown on the bare silicon wafer as a layer of insulation to prevent electric leakage. Then, the electrodes Mo and ${\mathrm{Al}}_{0.8}{\mathrm{Sc}}_{0.2}N$ were deposited by physical vapor deposition technology. The reason for selecting Mo as the electrode layer is that it can provide a good lattice match for the deposition of AlScN [51]. During each electrode deposition, reactive ion etching is employed to pattern the electrode, because the electrode coverage does not need to be maximum, resulting in the piezoelectric lamination shown in Figure 7b [24]. After the piezoelectric lamination is deposited and patterned, the upper and lower electrodes need to be connected in order to form the bimorph structure. Therefore, as shown in Figure 7c, the upper, middle, and lower electrodes are etched with through-holes to induce electrical signals. Precise etching is crucial to avoid Mo overetching, which could inadvertently induce signals in adjacent layers. After the above steps are completed, the Al/Cu is patterned after deposition, the purpose of which is to form a pad while extracting an electrical signal, as shown in Figure 7d. Next, the gap must be etched to outline the cantilever’s basic shape. Minimizing the gap width is crucial to prevent an enlarged leakage channel in the microphone, as shown in Figure 7e. The final step is to release the cantilever by deep silicon etching, removing the bulk silicon and thermal oxide behind it, resulting in the structure shown in Figure 7f, so that the cantilever is completely released and able to vibrate under sound pressure.

**Figure 8:**
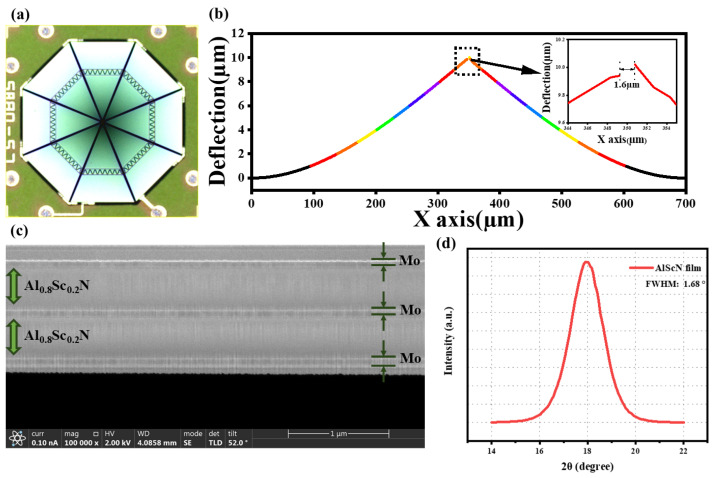
(**a**) Microscopical photo of the MEMS microphone. (**b**) Deflection of the cantilever beam. (**c**) FIB image of the cantilevers cross-section. (**d**) Rocking curves of the ${\mathrm{Al}}_{0.8}{\mathrm{Sc}}_{0.2}N$ film.

The Figure 8a shows the optical image of piezoelectric microphone, which is composed of eight cantilever beams and has a MEMS chip area of 900μm×900μm. Here, eight cantilever beams are connected in series, and the final output sensitivity will be approximately eight times that of a single cantilever beam. Due to the strict control of the deposition stress during the manufacturing process, the deflection of the cantilever beam is very small, as shown in the Figure 8b. The deflection of the cantilever tip by 10μm results in a leakage sound channel that is 0.6μm wider than the designed 1μm gap. Consequently, the microphone exhibits a lower cut-off frequency.

In Figure 8c, a focused ion beam (FIB) lamella was cut from cantilevers to investigate the microstructure of piezoelectric lamination. The cantilever beam is stacked with layers composed of Mo/${\mathrm{Al}}_{0.8}{\mathrm{Sc}}_{0.2}N$/Mo / ${\mathrm{Al}}_{0.8}{\mathrm{Sc}}_{0.2}N$/Mo, which is a typical bimorph structure. Figure 8c reveals that both the Mo and ${\mathrm{Al}}_{0.8}{\mathrm{Sc}}_{0.2}N$ films exhibited nearly ideal textured grain orientations.

The properties of the materials were comprehensively characterized. In the context of manufacturing high-performance MEMS devices, it is a common criterion that the Full Width at Half Maximum (FWHM) of piezoelectric films should be less than 2° [35]. The X-ray diffraction (XRD) rocking curves of the ${\mathrm{Al}}_{0.8}{\mathrm{Sc}}_{0.2}N$ films utilized in this study are presented in Figure 8d. For the ${\mathrm{Al}}_{0.8}{\mathrm{Sc}}_{0.2}N$ films with a thickness of 0.5μm, the measured FWHM is merely 1.68°. This result strongly suggests the viability of employing ${\mathrm{Al}}_{0.8}{\mathrm{Sc}}_{0.2}N$ as a piezoelectric material for the large-scale production of high-performance piezoelectric microphones.

In conclusion, within the scope of our research, a bimorph piezoelectric microphone incorporating ${\mathrm{Al}}_{0.8}{\mathrm{Sc}}_{0.2}N$ has been successfully fabricated. Subsequently, a comprehensive characterization of its crucial performance parameters will be carried out.

## 3. Results and Discussion

### 3.1. Frequency Response and Noise Measurement

**Figure 9:**
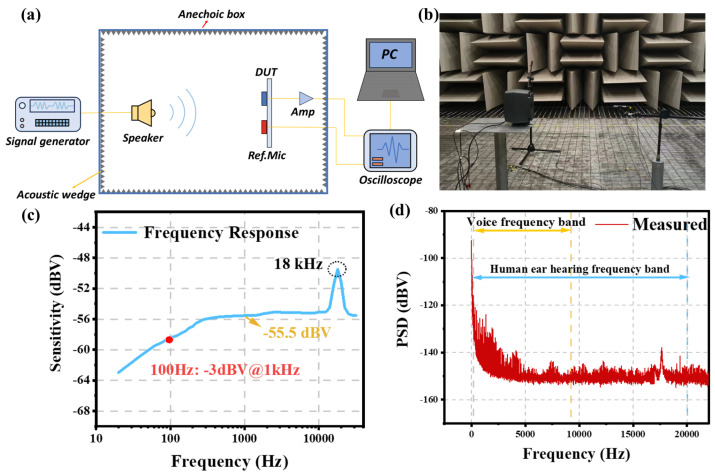
(**a**) The schematic of the frequency response of an acoustic experimental environment. (**b**)The frequency response acoustic experimental environment of the actual environment picture. (**c**) The frequency response diagram of MEMS microphone. (**d**) The measured noise of MEMS microphone.

To assess the acoustic performance of the microphone, relevant experiments must be designed. These experiments are conducted in the environment depicted in Figure 9a. The acoustic wedge is placed in the anechoic box, and it will absorb reflected sounds within environment [52]. This acoustic wedge can absorb sound throughout the entire frequency spectrum. Acoustic wedges exhibits more absorption on low-frequency noise, as given by:

(14) $$f_{co}=\frac{v}{4l}$$

where $f_{co}$ is the frequency, *l* is the length of the wedge, *v* is the speed of sound in the air (about 340 m/s). According to the actual situation, the length of the acoustic wedge is set to 1.8 m, which can absorb the sound above the 50 Hz frequency band.

Within the anechoic box, both the device under test and the reference microphone (GRAS 46AE) are positioned at an equal distance from the speaker (KEF Q100), and in a symmetric position. Initially, calibrate the sound pressure output and frequency response of the loudspeaker using a sound calibrator (BRUEL & KJÆR 4231). Subsequently, the calibrated loudspeaker is tested using the reference microphone. The signals from both the test device and the reference microphone are amplified and then transmitted to a data analyzer for further assessment. The actual layout photo is shown in Figure 9b.

The result is shown in the Figure 9c. The device exhibits a sensitivity of 1.68 mV/Pa at 1 kHz, which is remarkably high considering its small size and is attributed to the utilization of AlScN. Notably, the 1st resonance frequency of the device occurs at 18 kHz. This 1st resonance frequency lies well above the voice band (20 Hz–8 kHz), thus contributing to an excellent flat frequency response. The device also needs to be tested for total harmonic distortion (THD). The designs show a THD of less than 0.1% under the conditions of 1 kHz and 1 Pa RMS sound pressure excitation. As mentioned above, cantilever beams are bent due to residual stress and hence the gap between cantilever arrays will be enlarged. Given that the gap between cantilever arrays plays a crucial role in determining the sensitivity of microphones within the low-frequency range [24]. In a microphone, the frequency at which the sensitivity is 3 dB lower than that at 1 kHz is defined as the roll-off frequency, and the roll-off frequency of the device occurs at 100 Hz, which is the result of effective control of film deflection.

Utilizing the identical test environment mentioned previously, the device’s noise is assessed within an anechoic chamber to eliminate external environmental interference. Subsequently, the noise floor can be directly quantified utilizing the data acquisition software. The result is shown in Figure 9d, the low-frequency noise is prominent due to its difficulty in being effectively blocked. Specifically, near the frequency of 18 kHz, the noise attains a peak value, because the 1st resonance frequency is 18 kHz.

Within the human auditory frequency range (20 Hz–20 kHz), the A-weighted noise level of the device is −110 dBA, with an SNR of 54.5 dB. In the voice frequency band (20 Hz–8 kHz), the A-weighted noise decreases slightly to −111 dBA, resulting in an SNR of 55.5 dB. Under A-weighted calculations, low-frequency noise predominantly contributes to the noise floor, with component resonance noise having a comparatively negligible effect on the overall noise level.

### 3.2. Acoustic Overload Point and Test

As noted earlier, piezoelectric microphones, due to their operational principle, exhibit a higher acoustic overload point than capacitive microphones. This section aims to characterize the acoustic overload point of the fabricated microphones.

**Figure 10:**
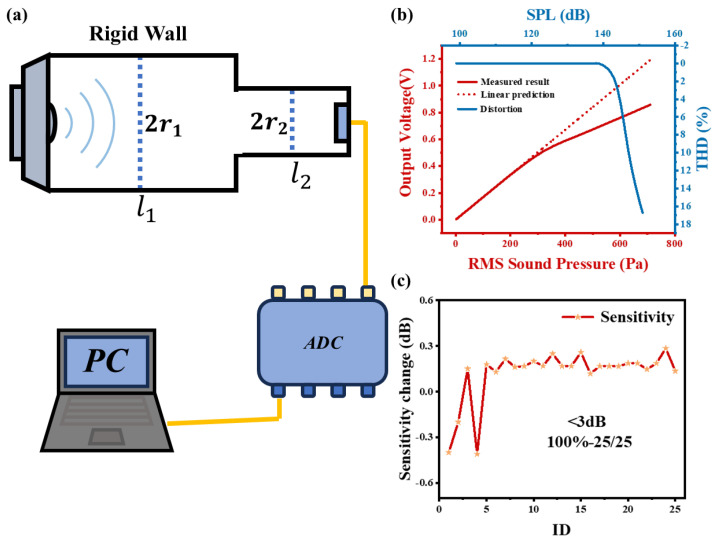
(**a**) Diagram of an ultra-high sound pressure level experiment. (**b**) The output voltage of the microphone at different sound pressure levels. (**c**) sensitivity of the microphone changes after a impact test.

Firstly, the acoustic overload point is defined as the sound pressure level at which the total harmonic distortion (THD) does not exceed 10% [24]. Typically, MEMS capacitive microphones have an acoustic overload point of approximately 125 dB SPL. However, achieving such high pressure levels in practical scenarios without compromising the authenticity of the sound source is challenging [53]. The sound pressure generated by the resonant coupling tube proposed by Oberst has the characteristics of high sound pressure and low distortion [54]. The specific tube is shown in the Figure 10a. The resonance condition of the resonant coupling tube is as follows:

(15) $$m\cos\left(kl_{1}\right)\cos\left(kl_{2}\right)-\sin\left(kl_{1}\right)\sin\left(kl_{2}\right)=0$$

where $m={\left(\frac{r_{1}}{r_{2}}\right)}^{2}$, *k* is wave number. According to the high sound pressure resonant coupling tube system described, it can realize the calibration of the microphone under test at 1000 Hz, 94 ∼ 171 dB SPL, and the total harmonic distortion of the sound pressure waveform is within 1.0%.

Within this testing system, the microphone was evaluated, and the resultant data are presented in Figure 10b. As the sound pressure steadily increases, the output voltage of the microphone experiences remarkable alterations. Significantly, when the sound pressure level exceeds 140 dB SPL, nonlinearity begins to manifest in the device’s response. Evidently demonstrated by the data, the acoustic overload point of the microphone attains 147 dB SPL, which far exceeds the acoustic overload points of all currently available MEMS microphones in the market [53].

Due to the need to improve the acoustic overload point of the device, the area of the device is reduced, and the SNR of the device is reduced. The manufactured piezoelectric bimorph ${\mathrm{Al}}_{0.8}{\mathrm{Sc}}_{0.2}N$ microphone achieves a sensitivity of up to −55.5 dBV and an SNR of 55.5 dB. The AOP of the microphone reaches 147 dB SPL. Owing to its elevated acoustic overload point and SNR, the microphone demonstrates an augmented dynamic range. Dynamic range is defined by the following formula:

(16) Dynamicrange=AOP+SNR−94

Table 2 shows the performance parameters of some microphones. The designed device has the highest Acoustic Overload Point (AOP) and dynamic range. In the future, the reliability of the microphone can be enhanced by further reducing its size.

Simultaneously, an impact test was carried out on the microphone, subjecting it to an acceleration equivalent to 10,000 times the acceleration due to gravity. Devices with a sensitivity change exceeding ±3 dB were considered damaged. The experimental data, presented in Figure 10c, show that all 25 test devices successfully withstood the shock test, achieving a 100% success rate. Moreover, the microphone has obtained IP58 certification in compliance with the IEC 60529 standard for water and dust resistance, which prominently demonstrates its excellent reliability.

## 4. Conclusions

First, the design concept of a single cantilever beam was discussed. A piezoelectric bimorph microphone made of eight triangular cantilever beams has been developed and analyzed. Addressing the limitations of piezoelectric microphones, ${\mathrm{Al}}_{0.8}{\mathrm{Sc}}_{0.2}N$ was employed as the piezoelectric material to enhance sensitivity, the adoption of a bimorph structure offers increased capacitance, thereby effectively reducing device noise. This microphone has a higher dynamic range and acoustic overload point compared to most products on the market. In subsequent designs, by further reducing the area of the cantilever beam, the resonance frequency of the microphone can be designed to be above 20 kHz, and the acoustic overload point can also be increased.

## Figures and Tables

**Table 1:** Some properties of common piezoelectric material.

Property	Symbol	Unit	PZT	ZnO	AlN	Al_0.8_Sc_0.2_N
Piezoelectric Coefficient	$d_{31}$	pC/N	−70	−5.74	−2.65	−3.89
Relative Permittivity	$\varepsilon_{r}$	/	1300	10.9	10.4	13.9
Loss angle	tanδ	/	0.03–0.05	0.01	0.002	0.0025
Young’s Moduli	E	GPa	56–98	208	338	230

**Table 2:** Properties of microphones designed by different manufacturers.

Name	SNR	AOP	Dynamic Range
Designed device	54.5 dB	147 dB SPL	107.5 dB
Miodrag, Germany [55]	66 dB	130 dB SPL	102 dB
MEMSensing, China	58 dB	130 dB SPL	94 dB
Infineon IM63D135A, Germany	63.5 dB	135 dB SPL	104.5 dB
Konwles SPH9855LM4H-1, America	66 dB	132.5 dB SPL	104.5 dB
Infineon IM69D130, Germany	69 dB	130 dB SPL	105 dB
Vesper 1010, America	60.5 dB	126 dB SPL	92.5 dB

## Data Availability

The original contributions presented in the study are incorporated within the article. For further inquiries, please direct them to the corresponding author.
